# Supplementary material for: scooby: Modeling multi-modal genomic profiles from DNA sequence at single-cell resolution
Source: bioRxiv. 2024 Sep 23:2024.09.19.613754. Preprint. [Version 2] doi: 10.1101/2024.09.19.613754 (PMC11429888; doi:10.1101/2024.09.19.613754)
Supplement: Supplementary file 1 [file media-1.pdf]

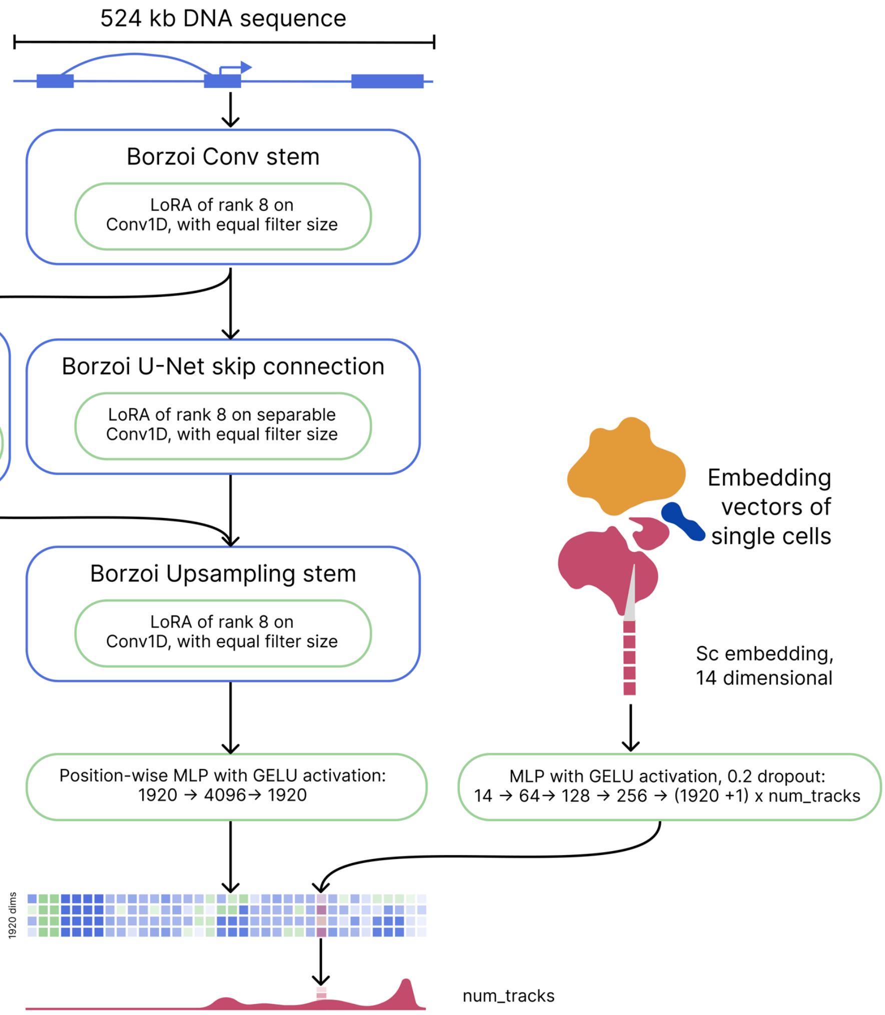

Extended Data Fig. 1: scooby architecture overview. Neural network diagram. Green boxes denote trainable parameters, blue boxes depict frozen (non-trained) model parts. 524kb of DNA sequence is processed by a LoRA augmented Borzoi stem with an additional MLP on the Borzoi embedding (left), whereas the single cell embedding is passed through a MLP (right) to predict the filter weights (red boxes) used to decode the sequence embedding.

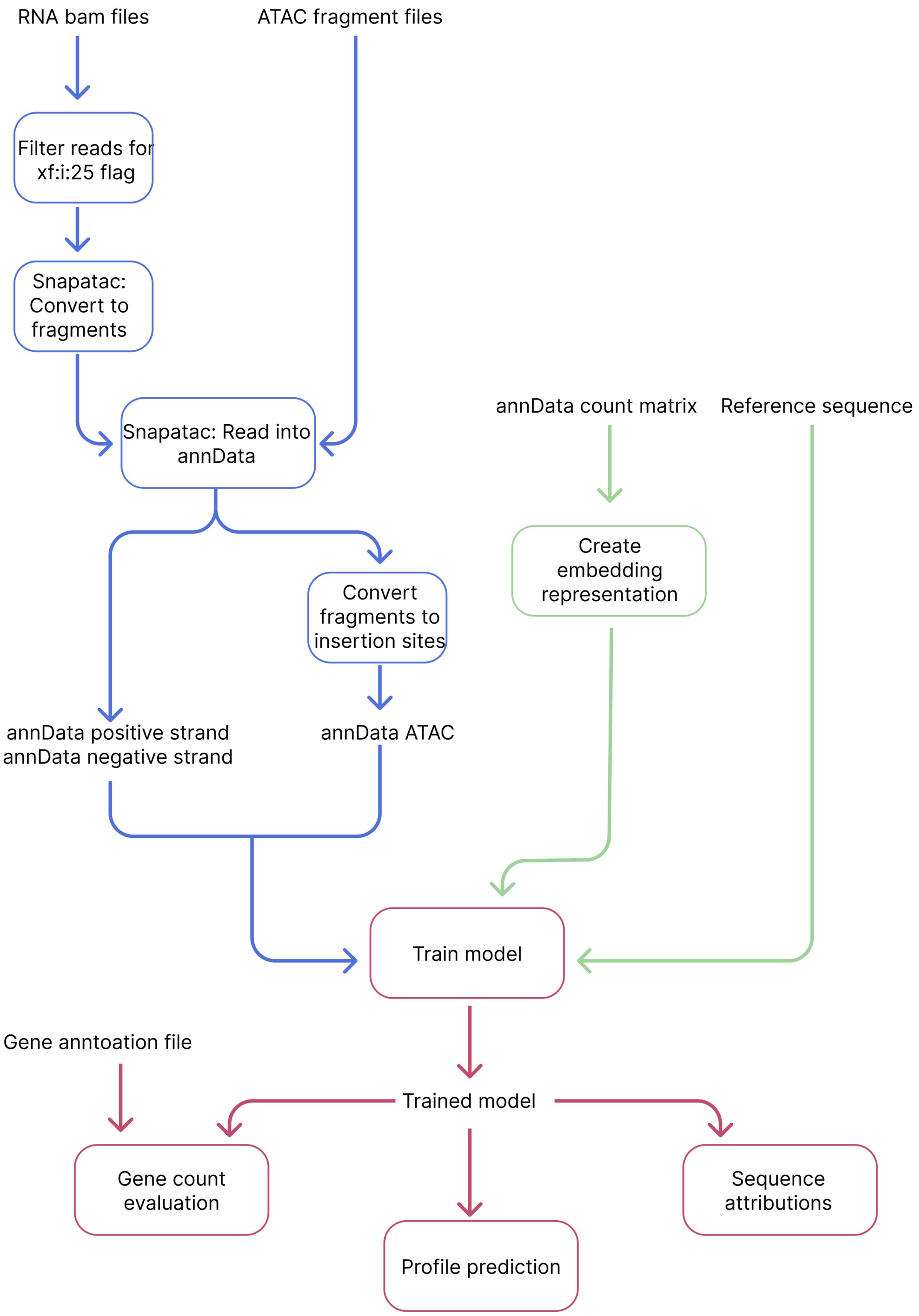

Extended Data Fig. 2: scooby framework flowchart. Diagram illustrates the workflow for generating cell embeddings and preparing RNA-seq and ATAC-seq data for training, including filtering, embedding generation, and coverage extraction.

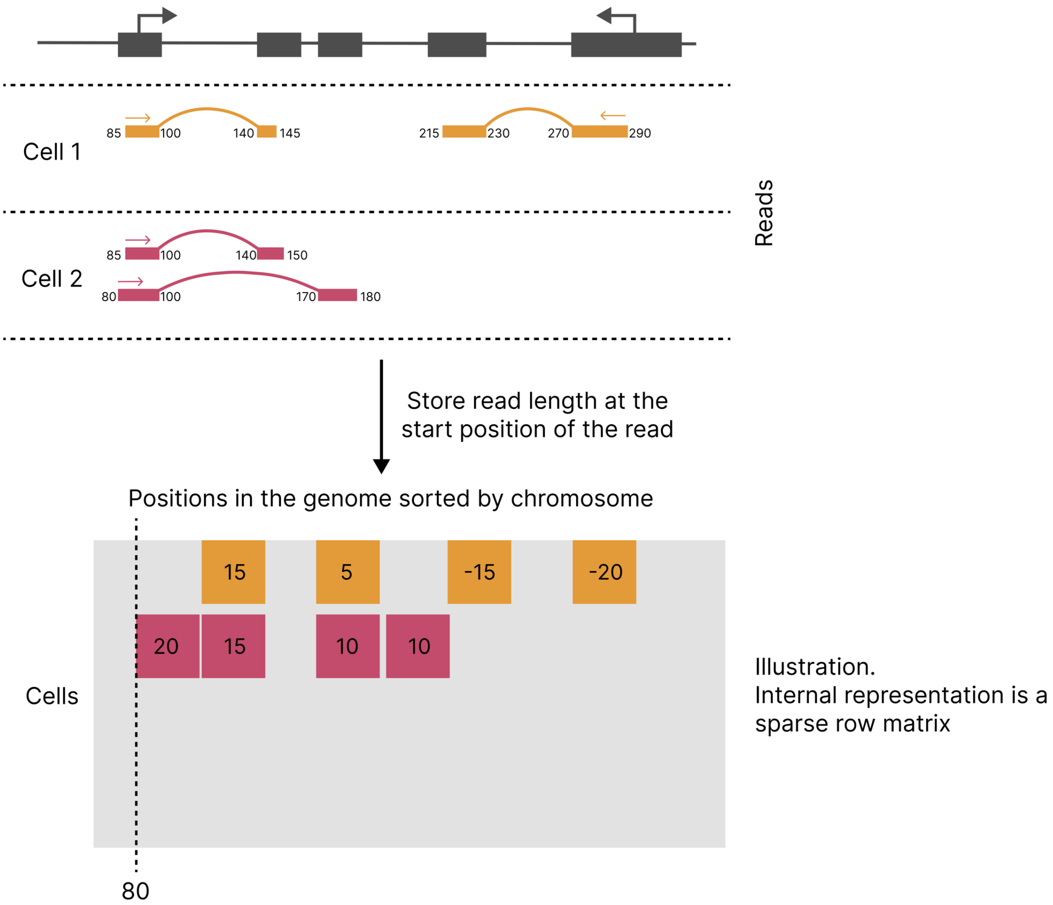

Extended Data Fig. 3: Memory-efficient storage of single-cell RNA-seq data using a modified snapATAC2.0 format. Each row in the resulting sparse matrix represents a cell, and each column represents a genomic position. Reads are stored at their start positions with values indicating read length. Split reads are stored as multiple entries. Negative values indicate reads mapped to the negative strand.

a **b**

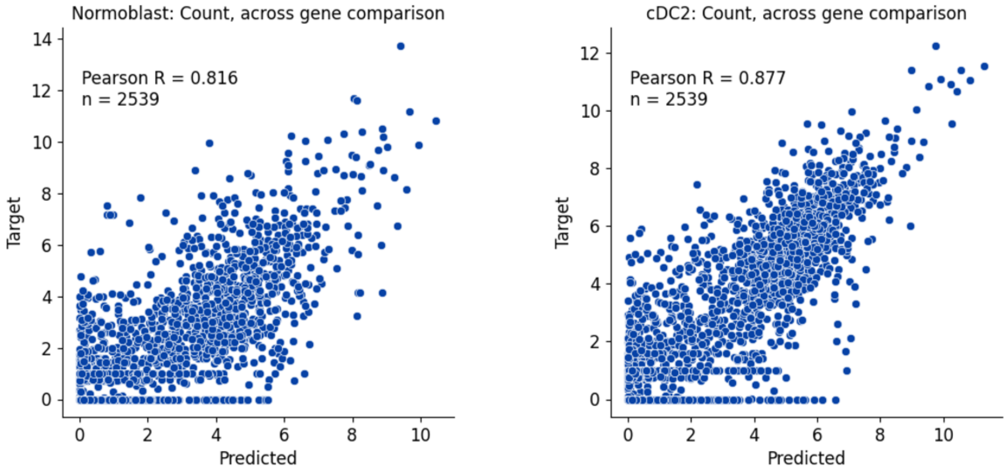

Extended Data Fig. 4: Log-transformed predicted versus observed counts of scRNA-seq reads overlapping exons for the worst (**a**) and best (**b**) predicted cell type.

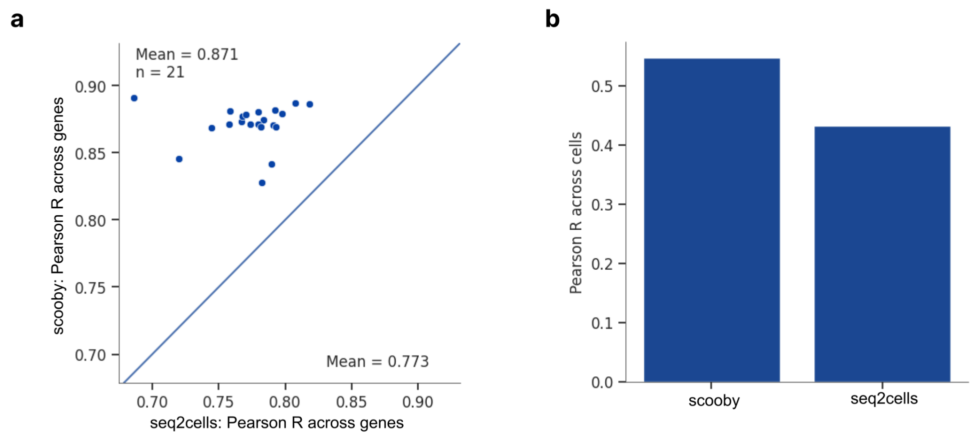

Extended Data Fig. 5: a, Across-gene Pearson correlation for all cell types comparing scooby and seq2cells. **b,** Between-cell-type Pearson correlation after subtracting gene and cell mean gene expression comparing scooby and seq2cells.

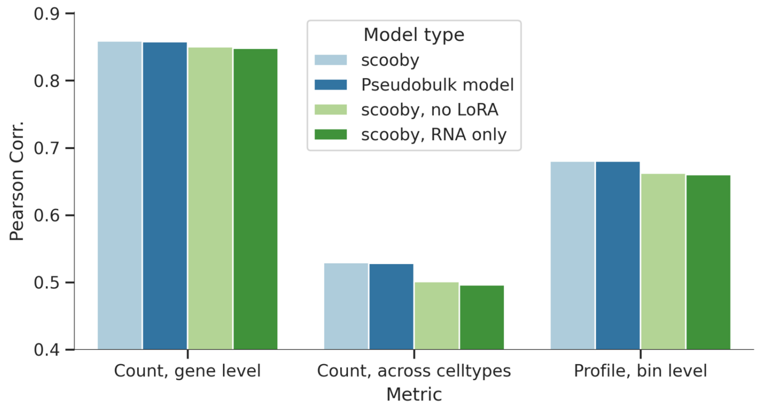

Extended Data Fig. 6: Performance comparison of alternative modeling approaches at predicting gene expression counts and binned profiles.

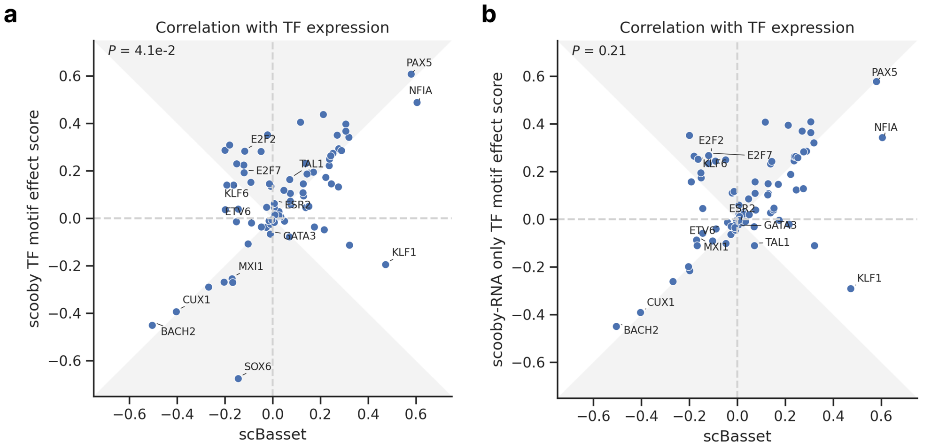

Extended Data Fig. 7: a, Pearson correlation of TF motif effect score with TF expression for scooby against scBasset. The gray area marks the zone of improvement. **b,** Same as **a** for a scooby trained on scRNA-seq only.

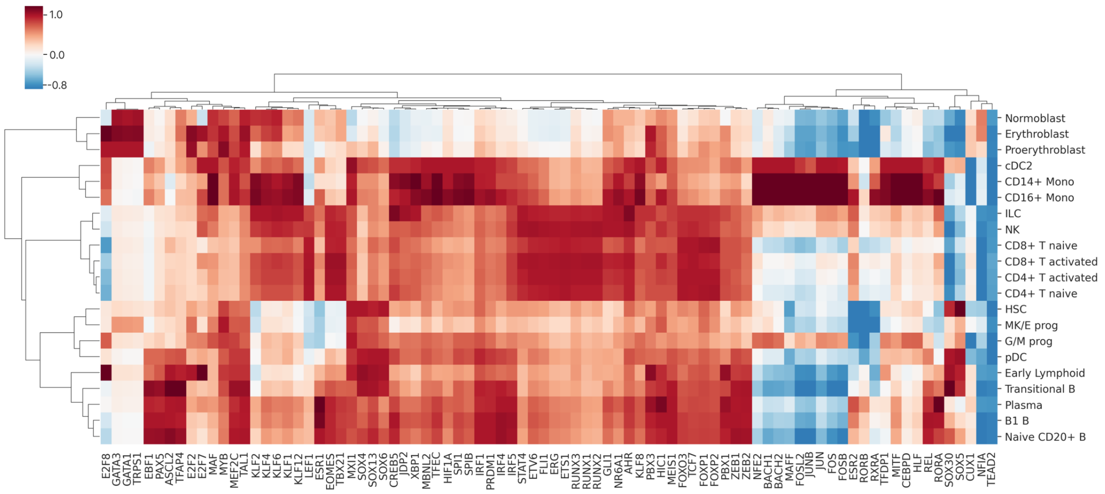

Extended Data Fig. 8: Heatmap of TF motif effect scores for differentially expressed TFs for all cell types. Genes and cell types are clustered according to their TF motif effect scores.

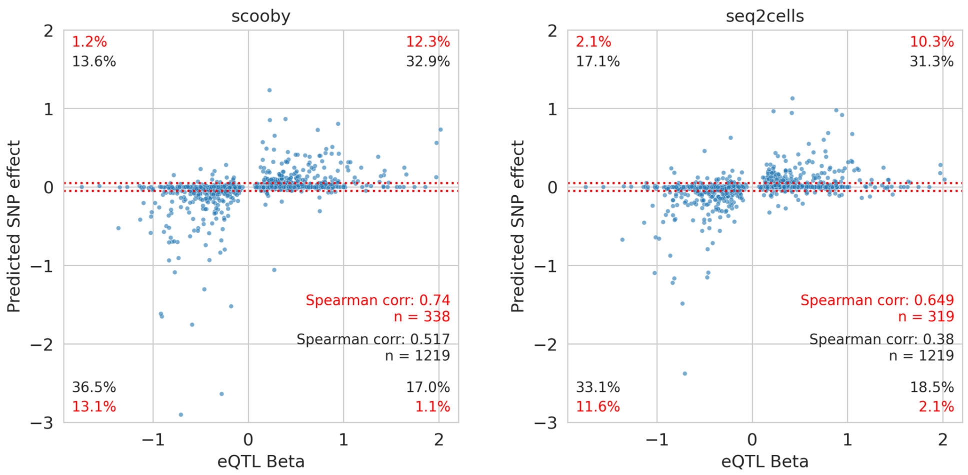

Extended Data Fig. 9: scooby (left) and seq2cells (right) predicted variant effects against reported eQTL effects on the common subset. Predicted aggregated effects (log-fold change) vs observed whole-blood normalized eQTL effect sizes. Red dotted lines mark thresholds below which predicted fold-changes are deemed negligible (absolute fold change 3.5%; matching the threshold by Schwessinger et al. for comparability). Percentages quantify variants within each quadrant: black - all variants; red - variants passing the 3.5% predicted effect threshold..

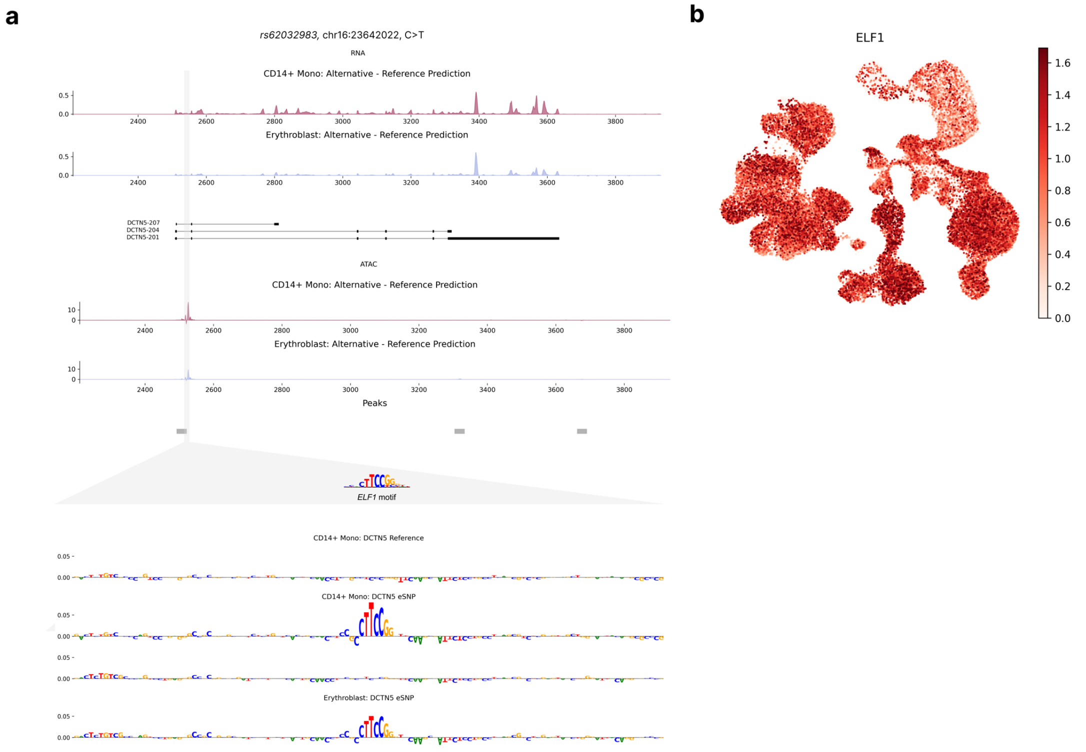

Extended Data Fig. 10: **a**, Predicted fold change in gene expression (top) and accessibility (bottom) between the alternative and reference alleles of variant rs62032983 in CD14+ Monocytes and Erythroblasts. Sequence attributions revealed the destruction of an *ELF1* motif to affect model outputs across cell types (Methods). **b**, UMAP of observed normalized *ELF1* expression levels.
